# British Association

**Published:** 1835-10-01

**Authors:** 


### British Association.

Our brethren of the Green Isle have had a rare intellectual banquet this year, and their numerous visitors from various parts of the world have had a double *feast*—spiritual and chylopoietic! Many a John Bull and Sawney M'Grig-gor—many a sprightly Frank and solemn German, will long remember the hospitality of Paddy's land! There appears to have been a remarkable and happy mixture of feasting, fun, and philosophy, on the banks of the Liffey, during the jubilee of science this year—such a combination as has not hitherto been seen, and such, we fear, as will not again obtain. The city of intellect in the North, and the great mart of commerce in the West, may hang their heads—the former has already been cast into the shade, and the latter may look to a similar fate, in the course of next August! But we must come to the philosophy, and lay aside the feasting.

Our indefatigable contemporary of Dublin has dedicated nearly one hundred pages of small type to an account of the proceedings carried on by the medical section alone—and from these ample details we shall draw largely in the present number, omitting, for the present, the foreign department, as not so interesting to the great bulk of our readers as the transactions of the British Association must necessarily prove.

*First day, 10th August. President, Dr.* Prichard.

Dr. B. Graves read an interesting paper on the internal use of chloride of sodium in fever, from which we make the following extract, as embodying the pith of the practical portion of the communication.

"With respect (said Dr. G.) to the time for its exhibition, and the cases to which it is adapted, the following remarks may be made. When the early stage of fever is past, when all general and local indications have been fulfilled, when there is no complication with local disease, when the patient lies sunk and prostrated, when restlessness, low delirium, and more or less derangement of sensibility is present, when the body is covered with maculæ, and when the secretions from the skin and mucous membranes give evidence of a depraved state of the fluids, it is then that the chloride of sodium may be prescribed with the most decided advantage. The mode in which I prescribe it is in doses of from fifteen to twenty drops every fourth hour, in an ounce of water or camphor mixture. How it acts I will not pretend to explain; it is sufficient to say, that there is no remedy from which, in such cases, such unequivocal benefit is derived. It operates energetically, though not very rapidly, in controlling many of those symptoms which create most alarm. It seems to counteract the tendency to tympanitis, to correct the fetor of the excretions, to prevent collapse, to promote a return to a healthy state of the functions of the skin, bowels, and kidneys; in fact, it appears admirably calculated to meet most of the bad effects of low putrid fever. To those who have witnessed its efficacy, it is unnecessary for me to say any thing. Of course it will fail, like all other remedies, when the disease has reached a certain point of intensity in individual cases. There is scarcely any acute disease, to which the human body is liable, which may not in some particular persons assume an intensity capable of baffling all the effort of medical skill. This, however, is no argument against the employment of a remedy of extensive utility and unquestionable value.

Although it is not my intention to give an account of what has been done in France with respect to the exhibition of this remedy, yet I may mention, that it has been extensively tried in fever by Chomel, and as I have learned with great success. This excellent physician is still, I believe, engaged in making further clinical experiments on the subject. In the *Gazette Medicale de Paris*, published on the 28th of last February, we have an account by Dr. Dor, of Marseilles, of several cases of typhus, in which the chloride of sodium was found beneficial in 1833. He attributes a more rapid amendment to the use of this remedy than I have ever seen follow from its exhibition, and he also asserts, that if not given with great caution, it produces a very tedious convalescence. In the latter remark, especially, I cannot concur; for all who witnessed this mode of treatment here, were struck with the security and quickness of recovery which ensued in those cases where it had been employed.—Perhaps, the precaution we adopted of always diminishing, as soon as possible, the strength and frequency of the doses, rendered the results in our hands more satisfactory than those obtained by Dr. Dor."

Dr. Graves concluded his observations by reading a letter from his colleague Dr. William Stokes, in which the use of this remedy is stated to have been followed by the most satisfactory results. It gradually but steadily removed all the bad symptoms, and in all cases the patients had most favourable convalescences. Dr. Stokes remarks, that all these cases recovered without any evident crisis.

### Dr. Houston.

The next paper, which was a very extensive one, exhibited the peculiarities of the circulating organs in living animals, by Dr. J. Houston. We can only spare space for one extract from this elaborate paper, and the discussions to which it gave birth. The following are the peculiarities which enable the whale and other air-breathing animals to dive into the recesses of the ocean, with respiration suspended for fifteen or twenty minutes, and yet without injury to the heart or lungs.

" And, first, as to the porpoise,—*(delph. phocœna.)* This animal in its organization resembles, most of any, the whale, of which, indeed, it may be considered a miniature. Its heart is very powerful, and the right ventricle bears a much greater proportional capacity and thickness, than that in land animals, a provision obviously connected with the greater resistance which the lungs occasionally offer to the passage of the blood. The venæ cavæ are also very capacious, and all the great veins leading to them are either remarkably numerous, large, and tortuous, or formed into reservoirs, capable of holding nearly all the blood of the body. When the abdomen of a porpoise is opened, the whole inner surface of the parietes, but particularly that along the back and sides, presents a livid hue—an appearance arising from the extraordinary congeries of veins, under the peritoneum, distended with dark coloured blood. When the canal in the vertebræ is exposed, all the space not occupied by spinal marrow or nerves will be found filled with large veins of remarkable transparency, devoid of valves, and arranged more like the sinuses of the dura mater, than like ordinary vessels. The branches and trunk of the vena azygos are also much bigger than usual. The vena hepaticæ are of great width, and with the other veins alluded to are capable of holding, without bursting, all that excess of blood which the asphyxiated condition of the lungs prevents getting forward. In addition, we find, that in this animal even the arteries in some places participate in the tendency to dilatability and tortuosity, so remarkable in veins. The intercostals, and other small arteries along the back and neck, take on, as shown in this beautiful preparation, a striking and unique plexiform arrangement, a fact first noticed by Tyson and Hunter. I have examined with care the blood-vessels inside the cranium, and have not observed that either the sinuses or veins, connected with the brain, differ from those of terrestrial animals—a circumstance which deserves to be noticed, as it would seem to indicate, on the part of the Creator, a watchfulness in protecting the centre or source of life in the animal, from the injury which must accrue to it, were the blood to accumulate about the organ in inordinate quantity."

" It will be seen by these preparations, that, in the seal, the dilated condition of the veins is even more remarkable than in the porpoise. Those in the spinal canal are of extraordinary dimensions: the posterior and lateral parts of the neck, and also the back, are covered with a network of veins, which, when injected, cover over and hide from the view every other texture. An arrangement such as this is not found in any terrestrial animal; and, as far as I know, has never been described by any author. The liver is, as it might be expressed, scooped out into one vast reservoir; the hepatic veins forming bags rather than vessels, capable in themselves of holding nearly all the blood of the body. In this preparation, the contrast between the vena porta and hepatic artery, which are not one-third the size of the same vessels in the human body, and the enormous hepatic veins, whose diameter is greater than that of the right auricle of the heart in man, is singularly striking. The veins in the abdomen covering the lumbar muscles, and those on the surfaces of the kidneys are so capacious, that when filled with injection the parts beneath them cease to be visible."

"In the animals, before spoken of, whose watery habitation is more extended and deep, and whose exercises, under water, in search of prey, or in evading enemies, are more prolonged, we find the provision of reservoirs more enlarged and more generally extended throughout the venous system of the body.

As we might be prepared to expect, a provision such as that which exists in the otter, is present also in diving birds, in accommodation to their habits of remaining under water in search of the fish on which they prey."

"To appreciate the necessity for a provision in diving animals, such as that, the exposition of which we have been engaged in, it is only necessary to bear in mind, that such is the difference between the effects of the venous and arterial blood on the functions of the body, in warm-blooded animals, that it is essentially necessary they should be confined to their own respective vessels, and not mixed up with each other, as has been supposed by some to occur in divers. The blood of the jugular vein, sent back directly to the brain by the carotid artery, (an experiment of the celebrated Bichat), caused instant death to the animal."

"The reservoirs then, which we have seen to exist in the course of the veins leading to the lungs and heart in aquatic mammalia and birds, by allowing a temporary resting-place for the blood when stopped in its free course during the obstruction to respiration which occurs in the act of diving, are perfectly compatible with the established laws which regulate the movements of the fluids in living bodies, and absolutely necessary towards the existence of these animals under the circumstances in which they are placed."

An animated discussion followed, and was chiefly sustained by Mr. Hargrave, Professor Alison, Dr. Harrison, Mr. Adams, Dr. Hart, Dr. Jacob, Dr. Williams (London), and Dr. Corrigan.—For the remarks of these gentlemen we must refer to the Dublin Journal itself.

*Second day, Aug. 11th. Dr.* PRICHARD *in the Chair.*

The motions and sounds of the heart occupied the whole of this sitting. A committee had been appointed to experiment on this subject, and having made their experiments, and drawn up their report, they presented them to the meeting. We cannot notice all, or many of the experiments performed by the committee, but only one or two, to elucidate the inquiry.

EXPERIMENTS ON THE MOTIONS OF THE HEART.

"*Experiment* 1. A calf, two days old, having been secured on its back, and prepared as above described, the sternum and a portion of the ribs on both sides were removed, when the following motions were observed. The heart was beating strongly, at the rate of 144 pulsations in the minute, but in a short time fell to 80. While still enclosed in the pericardium, the heart was observed to have a slight libratory motion on its longitudinal axis, which motion, it may here be remarked, may assist in explaining the phenomenon of *frottement* in disease. On cutting open the pericardium, and turning it aside, both the auricular appendices were seen to project with a rapid motion upwards, or towards the place of the sternum, and immediately afterwards to recede. When coming forwards, they were swollen and soft to the touch; when receding they became hard to the touch, were diminished in size, and flattened. Immediately after the recession of the auricular appendices, the ventricles with a rapid motion assumed a somewhat globular form in their middle part, which projected towards their sternum, and their apex at the same time was pushed considerably in the same direction. During their continuance in this state, the ventricles were hard to the touch, and if grasped by the hand, at the commencement of the movement, they communicated a shock or impulse, and separated the fingers. When the ventricles had remained for a short time in the state just described, they suddenly sank downwards or towards the spine, and became elongated, broad and flat, and soft to the touch.

This succession of motions having been observed for some time, a small glass tube was introduced through a puncture into the left auricular appendix, and the blood was seen to rise in the tube during the recession of the appendix, and to subside during its upward movement. A similar tube was introduced through a puncture in the right ventricle, and a jet of dark-coloured blood was thrown forth during the globular and hardened state of the ventricles, and subsided when they became flattened and soft. A puncture was made in the pulmonary artery, close to the ventricle from which it arises, and through it a stream of blood issued synchronously with the jet from the tube in the right ventricle. A tube having been introduced through a puncture in the left ventricle, and one of the mesenteric arteries having been exposed and opened, the jet from the ventricles was observed to precede the jet from the arteries, by an interval easily appreciable. The femoral artery was opened, and a similar observation was made as to the interval between the jet from the left ventricle and the jet from that artery. Previously to opening the chest, the committee had satisfied themselves, that the beat of the heart, felt through the sternum and cartilages of the ribs, preceded the pulse, felt in arteries at different distances from the heart, by intervals of time which were proportioned to those distances: and they were also satisfied, that the jets of blood from the mesenteric and femoral arteries were synchronous with the pulses felt in those arteries.

*Experiment* 2.—In a calf, prepared as the former had been, and placed on its right side, a portion of the ribs on the left side was removed, the sternum and part of the cartilages on that side being left in their natural position, and the pericardium was opened. It was now seen that when the ventricles assumed their hardened state, their apex, and a considerable portion of their anterior surface were closely applied to the sternum, and when the hand was interposed between the latter and the surface of the ventricles, a strong compression was exercised on the fingers during each approach of the ventricles to the front of the chest. When the ventricles were in their softened state, their interior surface, by which is meant the one corresponding to that called anterior in the human heart, was sometimes in contact with the sternum, and sometimes removed to a little distance from it, and from the contemplation of this, and the preceding experiment, the committee were satisfied, that the situation of the heart in the thorax is affected by the position of the body, as has been observed by others; for instance, that in the recumbent state, on the back, the heart recedes somewhat from the sternum: if the individual lie upon the face, the anterior surface of the ventricles is in constant apposition with the front of the chest, the pericardium of course being interposed. The yielding texture of the lungs, and the mode of attachment of the pericardium and the great vessels, are such, as to allow the gravitation of the heart to influence its position in different postures of the body. These experiments were repeated on different subjects, and the observations recorded above were confirmed."

For the experiments on the sounds of the heart we must refer to the Dublin Journal, contenting ourselves with the *conclusions* to which the committee came from the said experiments.

"From the experiments on the sounds of the heart, it appears to follow: 1. That the sounds are not produced by the contact of the ventricles with the sternum or ribs, but are caused by motions within the heart and its vessels. 2. That the sternum and front of the thorax, by their contact with the ventricles, increase the audibleness of the sounds. 3. That the first sound is connected with the ventricular systole, and coincides with it in duration. 4. That the cause of the first sound is one which begins and ends with the ventricular systole, and is in constant operation during the continuance of that systole. 5. That it does not depend on the closing of the auriculo-ventricular valves at the commencement of the systole, because such movement of the valves takes place only at the commencement of the systole, and is of much shorter duration than the systole. 6. That it is not produced by the friction of the internal surfaces of the ventricles against each other, as such friction cannot exist until the blood has been expelled from the ventricles, whereas the first sound commences with the beginning of the ventricular systole. 7. That it is produced either by the rapid passage of the blood over the irregular internal surfaces of the ventricles on its way towards the mouths of the arteries, or by the *bruit musculaire* of the ventricles, or probably by both these causes. 8. That the second sound coincides with the termination of the ventricular systole, and requires for its production the integrity of the semilunar valves of the aorta and pulmonary artery, and seems to be caused by the sudden check given by the action of these valves to the motion of the columns of blood driven towards the heart after each ventricular systole by the elasticity of the arterial trunks.

The Committee wish, in concluding this report, to express their opinion, that although much light has been thrown on the subject of the Motions and Sounds of the Heart, by recent investigations, here and elsewhere, the nature of the inquiry is such as renders it difficult in many instances to arrive at satisfactory conclusions. They also think that the subject, from its importance, whether in a practical view, or as an object of philosophical inquiry, is deserving of further investigation."

As might be expected, a long discussion ensued, in which Dr. Willams, of London, and Dr. Corrigan, of Dublin, were the most prominent orators. Dr. Corrigan candidly confessed that he was wrong in some of his first conclusions, and was convinced that his character would not suffer by this avowal. We are sure it will not; but, on the contrary, be raised by such honorable and honest procedure. The thanks of the meeting were voted unanimously to Dr. Williams.

*Third Day. Dr.* Prichard *in the Chair.*

Dr. M'Donnell, of Belfast, read a paper this day on the action of the heart and pulse. Our author has some claim to antiquity, as well as to "priority, in such observations, having commenced as early as 1784"—that is, rather more than half a century back.

"He finds that in lying, sitting, or standing, there are three distinct numbers in the pulse, any one of which being given, the rest may be discovered by inference. This variation amounts generally to twelve, fourteen, or sixteen beats per minute, as its normal state, and therefore, that all observations of the number of the pulse, which have been made without reference to this principle, must be considered as nugatory, unless it be implied that the person was in the *horizontal position* when the observation was made. This rule for reducing the number of the pulse to a regular standard applies to health, but not precisely to disease; the effects of posture must be investigated separately in each disease.

The *differential pulse* appears to be confined to man. It is not observed in brutes, probably because, from their form, their posture may be considered as always horizontal; but when placed erect this peculiarity appears also in them.

The variation, in the human species, is at its maximum in tall and feeble subjects, particularly in convalescents from typhus; the minimum is generally found in children. These facts lead to the supposition, that this phenomenon is connected with some hydrostatic law, and not depending entirely on vitality. This, however, is merely thrown out as a conjecture, and requires further investigation. But in whatever manner it may be considered, it is plain that in all attempts to ascertain the effects of remedies, as well as of natural causes, due allowance must be made for these fixed differences produced by posture. What avails it to say that a medicine, or venesection, or heat, or cold, or a thousand other natural causes, raise or depress the pulse by four, six, or eight beats per minute, when the mere change of posture would raise or depress it twelve, fourteen, or sixteen per minute, and this merely in health, for in disease the differential pulse is often double this proportion.

In tracing the connexion between the pulse and respiration in man and quadrupeds, he finds that it ranges in health from four to six pulses for one respiration. This he considers a new and material fact; for if it be established by further observation, that this is a general law, we shall be able to infer the pulse from the respiration, and *vice versâ*. This may be of advantage in enabling us to ascertain the number of the pulse in ferocious animals which we dare not touch, as well as in man during action or progression.

There is a coincidence between the number of pulses and steps in walking, at the common rate of progression in man, that is very remarkable and has not been hitherto noticed. His breathings are also singularly proportioned to his steps, so that it is easy to deduce these numbers from each other. But in hard labour or violent muscular exertion, as in running or ascending heights, the proportions are greatly altered. The same thing occurs in many forms of disease. There is reason to believe, that the carbonization of the respired air, has a great influence in all those cases where the number of respirations is greatly disturbed.

Dr. M'Donnell finds that the number of respirations, and by inference the number of pulses, are much the same in passing over the same space, whether we run or walk, *i. e.* they depend as much upon the space traversed as on the time. Thus he finds, if he walks 1000 yards in ten minutes or in eight, or runs over it in five minutes, the number of breathings are nearly the same. It is to be observed, however, that this rule does not apply to small portions of space, such, as 50 or 100 yards. These facts, he thinks, are all complicated with carbonization and muscular motion, so as to require separate investigations.

In quadrupeds, especially when trotting or cantering, he has found that the steps, divided by the respirations, never give any fraction in the quotient, *i. e.* that these are universally proportional without any deviation. In man this does not occur, a circumstance which may arise from some peculiar anatomical or physical law in the connexion between the respiratory and muscular construction of these animals."

"While engaged in these enquiries, about thirty years since, he had found that the pulse in the arteries of the fœtus, before it breathed, was *slower* than in those of the mother. He had found also, that if the child, when born, remained for some time without breathing, the pulse continued slow during that interval, and became accelerated only at the instant it took in its first breath. This fact appearing to him new, he had investigated the circumstance in the cow, and finding the phenomena similar, he had communicated his observations to Dr. Clarke, Dr. Labatt, Dr. Stokes, and Dr. Douglas in Dublin, none of whom had ever noticed it in any other. Dr. Jefferay mentioned it in his lectures, and in his 'Observation on the Heart and on the Peculiarities of the Fœtus,' and thought it might hold true of quadrupeds, and of all warm-blooded animals. He thought it probable that the fœtus before respiration was in the condition of a cold-blooded animal, and partook of that slowness of the pulse which characterizes the tribe."

Dr. Collins observed that, from the experiments he had made on the subject, he had come to a very different conclusion. "He had invariably found the circulation of the fœtus much quicker than that of the mother." In this observation we concur. Why should the circulation be quicker in children than in adults, if it were slower in the fœtus than in the child. Perhaps the respiration may account for this difference.

Here the discussion turned on the interminable subject of the "sounds and motions of the heart," to which, we think, a great deal too much importance has been attached, considering the vast field of pathology and therapeutics which lies half cultivated. Mr. Carlile took an active part in this renewed discussion, which, however, was terminated rather abruptly at last by Dr. Collins, who rose, and stated that the discussion was becoming too protracted, as very many papers of importance were to be read before the Committee." Dr. Harrison then brought forward a paper on—

"Certain Bones found in the Hearts of some of the Ruminantia."

After some general observations on the wisdom of the Divine Artichect, as evinced in the construction of the various circulating organs—"all having a common type (though varied *ad infinitum*) and bearing the impress of one common hand—so wonderfully contrived, in every part, that the most extraordinary ingenuity could not make in them a single improvement," Dr: H. observed :—

"Of all animal, the best specimens of these bones were to be found in the ox. It has been stated (said Dr. Harrison) by some, that they are not to be found in the young animal, but this is a mistake; here is the heart of a calf three weeks old, and you can perceive how rapidly the development of these bones is going on. Here is a cartilaginous mass in the situation of the larger bone, and you can see a nucleus of bone in the centre of it very plainly. This shews clearly that those ossific deposits are not, as Carus thinks, accidental, but are the result of a natural process.

From the uniformity of the size and structure of these bones, and from the numerous dissections I have made, I am led to consider that they serve the following purposes. The heart of the ox is very peculiar in its structure; its cavities are very small, its muscular parietes very thick, and of enormous power. Now, the muscular structure, if examined, will be found to stop short at the tendinous insertions of the aorta, and, consequently, it leaves that portion of the vessel which corresponds to the semilunar valves in a weak and unprotected state. I conceive, therefore, that one of the purposes which these bones serve, is to strengthen the roots of the great vessels in these situations, and defend the opening of the aorta from the effects of violent contraction. In the second place, this bone (the larger one) affords a degree of protection to the auricle, which has a larger cavity, but is not so strong as the ventricle; and in addition to this, affords a fixed point for the contraction of the muscular fibres during the auricular systole. It may also serve to prevent that cavity from being completely closed. Thirdly, it gives strength and attachment to the mitral valve, which is fixed to its upper border. Again, when we consider the size and calibre of the aorta in the ox, and the extraordinary thickness and elasticity of its parietes, we shall readily see the necessity of having some provision to support the pressure caused by the returning columns of blood. Accordingly, when we examine the aortic sinuses we find, that of two of them, the floors are formed by these bones, in addition to the ordinary structure. The third sinus of the aorta, which is deprived of this protection, is defended by a peculiar provision, consisting of a remarkably fatty deposit of a hard and firm nature, and which is found in the youngest animals, even in those which are very lean. The sinuses of the pulmonary artery are also defended by this fatty substance; in fact, it appears to be their principal protection, for when dissected off, the walls appear so weak, as to be scarcely capable of bearing the impetus of the returning blood. The semilunar valves in the calf and ox are also very beautiful, and the corpora Aurantii highly developed, so as to afford good grounds to think, that they are intended chiefly for the support of the valves along their free margin."

Dr. Harrison exhibited some beautiful specimens of these bones. No discussion followed.

### HYDATIDS IN THE OMENTUM OF THE AXIS DEER. DR. HOUSTON.

Our Dublin contemporary considers this paper so valuable, and the drawings so important, that he has reserved it for his next number, giving, at present, a mere outline of the paper.

" The paper contains a description of several hydatids, but more especially the *cysticercus tenuicollis*, of which a great number, of large size, were found in the omentum of an axis deer, which died in the Zoological Gardens, at Dublin. The principal object of the paper is to determine the pathological changes, which hydatids and other parasitical animals undergo, and their relation to malignant or tubercular diseases. The degeneration of hydatids through all their changes is described, and the phenomena of degeneration are traced by Dr. Houston to a different source and termination, from that supposed by other authors to belong to them. The following extract from the paper, will give some idea of Dr. Houston's views on the subject. ' With respect to the question, whether the disease in the cyst proceeds and becomes the cause of death in the hydatid, or that the animal, after passing into the state of death, and thence acting as a source of irritation on the investing membrane, gives rise to those changes in that texture, which have been just described, it is very probable that the latter view of the case is the correct one: viz. that the worm having arrived at a certain age dies; that it then comes to act as a foreign body; and that all the subsequent changes which take place in the cyst are merely the consequences which flow therefrom. The numerous and singular differences in the condition of the hydatid tumours, from that state in which they exist as translucid and living bodies, to that in which they appear in the form of small bone-like particles, would seem to indicate, that a long period had elapsed since the development of the first series of hydatids in the omentum, and that these having passed away, were succeeded by others, which, in their turn, gave place to ternary and quaternary formations, each equally subject to decay, and running by the same processes into the same ultimate states of degeneration. In fact, the animals might be said to have established, in the omentum, a sort of colony—propagating their young, passing through *' les quatre âges de la vie,'* and finally converting their habitations into sepulchres, where their bodies passed into complete decay, without farther damage to the surrounding parts. There is most probably with these, as with all animated beings, a limit to the term of their existence; a period beyond which they are fated not to retain the condition of vitality.' "

### ENTOZOA IN HUMAN MUSCLES. DR. HARRISON.

Dr. H. exhibited an enlarged view of a human biceps muscle, the body of which was dotted over with white oval specks, which, on examination by the microscope, were found to consist of a semi-transparent cyst, of elliptic form, in the interior of which lies, coiled up, a minute worm. These worms have been frequently observed to move about after being dislodged from their cysts. They are of a pyriform shape, and seem to have a transverse opening at their larger extremity. Dr. H. never found these worms in any of the involuntary muscles. They occur chiefly in the muscles of the back and loins. The subjects in which they were found, were all scrofulous and emaciated. Dr. Roe, of Cavan, stated the following curious case, though it did not seem to bear very particularly on the subject of Dr. Harrison's paper.

" He was called some time back to visit the daughter of a farmer residing at Drum, in the county Cavan ; the girl had been attacked several days previously with inflammation of the thigh, and at the time of his arrival was labouring under severe symptomatic fever with delirium. The thigh was tense, red, and shining, enlarged to nearly twice its natural size, and extremely painful. No cause could be assigned for the disease, and he was informed, that until the occurrence of the present attack, she had always enjoyed excellent health. Having ascertained the existence of a collection of matter under the fascia, he made an incision, and evacuated a bowl-full of pus, mixed with what he considered to be clots of blood. His attention was not directed to the contents of the abscess at that time, besides the room was dark, and he was anxious to give vent to the pus as quickly as possible, as the patient was extremely restless. On emptying the matter from the bowl on a clean flag outside the door, the girl's mother was surprised to find among it a leech coiled up, quite alive and moving actively. She immediately brought the leech to me and it continued to live for several days afterwards. On inquiring minutely into the history of the case, I found that some days before she first complained of the limb she had been gathering water-cresses in a ditch and had felt hurt in or about the ankle of the inflamed limb, but did not pay much attention to it at the time. On examining the ankle I found a triangular cicatrix such as that which might be produced by a leech-bite. This fact would seem to prove that such animals can enter, burrow in, and preserve their vitality in the soft parts of the human body."

Mr. Crampton mentioned that several of the deer in the Phœnix Park had manifested symptoms of delirium, and were obliged to be shot. On dissection the tracheæ were found filled with worms. They were pendulous, and attached by the head, which resembled that of a leech. The brain was examined, but no disease found there. After some desultory conversation, the meeting broke up.

*Fourth Day. Dr.* Prichard *in the Chair.*

Dr. Roupel this day detailed some experiments on poisons, and exhibited drawings illustrating the effects of these on various structures. Our readers are aware that in vol. 20, page 62, &c. we gave an account of Dr. R's work on poisons. We deem it not necessary, therefore, to dwell farther on the subject in this place.

Dr. Alison on the Vital Properties of Arteries leading to Inflamed Parts.

It appears that the distinguished Professor of Edinburgh has been engaged for some time past, in endeavouring to form an estimate of " the physiological principle of spontaneity of movement in animal *bodies*; in other words, of the movements which occur in these bodies, independent of their living solids." There appears some mystification in this passage. By animal *bodies* we apprehend that the Professor means *fluids;* for of spontaneous movements in muscles, for example, there can be no doubt.

" Two branches of this inquiry are particularly deserving of attention. 1st. Whether the phenomena of inflammation can be explained on the supposition that the only vital principle is the action of the heart and vessels. 2ndly. If the movements of the blood through the lung can be explained on this principle. Dr. Alison thought these questions must be answered in the negative.

The first series of experiments made by Dr. Alison went to prove that the vital power of tonic contraction (to which Dr. Parry has given the name of tonicity, and which determines the degree of contraction in arteries after death) becomes diminished in arteries going to inflamed parts. Dr. Alison here detailed an hydraulic experiment which was made on axillary arteries of a horse, the one leading to sound, the other to inflamed parts, by means of two tubes furnished with stop-cocks; the rise of the water in one of the tubes being the measure of the distention of the artery. By these it was found that the re-action of a sound artery raised the level of the fluid sixteen lines, while that of the diseased artery raised it only ten lines; thus proving that the sound artery was capable of greater distention than the diseased. As this experiment might be objected to, as being performed ten hours after the animal's death, the axillary arteries of another horse were experimented on half an hour after the animal died. The subject of the experiment had laboured under violent inflammation of the knee joint, for which blood had been copiously extracted. On measuring the arteries of the diseased and sound limb, those of the latter were found much larger. The diseased arteries were also found to become enlarged as they approached the inflamed part. A portion of artery which was examined, measured 7-8ths of an inch at its upper or smaller extremity, and 12-8ths at its lower. In this experiment the artery of the sound limb expelled 1¾ inches of water, while the artery of the inflamed limb expelled only 1½. In another experiment made by Dr. Spittal, the greater dilating power of the sound artery was also observed; the difference between the contracted and dilated state of the sound artery being 8-24ths, of the diseased artery 6-24ths.

From these experiments, it results, that the arteries leading to inflamed parts become relaxed and weakened, transmitting the impressions given by the heart with less modification, and having less power of contracting on the blood. This being established, the question is, whether this state of the arteries is an adequate cause for the phenomena of inflammation, that is, whether inflammation consists in this condition of the vessels? He thought this question required further investigation. Two changes occurred in all cases of inflammation, a retarded movement of the blood in the vessels of the part, and an increased movement in the surrounding vessels. If we suppose that inflammation consists in an altered action of the vessels of the inflamed part, we make no attempt to explain the difference between inflammation and simple congestion. This subject requires a vast number of experiments for its elucidation."

There is surely not much novelty in this doctrine. Every one knew that the vessels leading to inflamed parts were dilated—and it is nearly half a century ago that numerous experiments were made in Edinburgh (Lubbock, Allen, Wilson), to prove that the capillaries were weakened and dilated in inflammation—and consequently that the circulation was actually slower in inflamed than in sound parts. We have always considered this as the true doctrine of inflammation, and we are glad to see so able an advocate of the same in the person of Professor Alison. The doctrine of "increased action," in inflamed parts, is apparently supported by the utility of local evacuations from the vessels of the part. But it is to be recollected that vascular distention produces *irritation* in the nerves and muscular fibres of the portion inflamed, and that the removal of this distention by detraction of blood, takes off the source of irritation and pain.

"The next part of Dr. Alison's paper contained some notices on the cause of death in cases of asphyxia.* He commenced by stating that it was well known that the admission of air into the lungs was absolutely necessary for the prolongation of life; that when air was excluded, the power of the heart became inadequate to the transmission of the blood, which, under such circumstances, could no longer pass through the lungs. The question then was, in what manner respiration exercised this auxiliary power? He referred to the doctrines of Haller, Bichat, Goodwin, and others, and stated that the theories of the two latter being found erroneous, the doctrine of Haller had been again revived. The best mode of bringing this doctrine to the test was, to shew what occurs in an animal breathing a gas which contains no oxygen; where the mechanical change is

* We can only afford space for a short extract containing Dr. A's conclusion.

going on, but the chemical is suspended. He, therefore, thought, that by confining an animal in azote until its breathing became laboured, and then taking it out and killing it immediately, he might have an opportunity of ascertaining whether the blood passes through the lungs, as in a case where the chemical change is going on. A rabbit having been confined in azote, was taken out, and its sensibility destroyed instantaneously by a blow on the head. It never appeared to inspire after the blow was struck, but was slightly convulsed. On opening the chest, Dr. Alison was surprized to find that the contractions of the heart were extremely feeble. The right side of the heart was distended with blood, and the quantity of blood, procured by puncturing the pulmonary artery, was ten times as much as could be got from the left side of the heart. In the right side of the heart there was a faint pulsation, which continued for a short time ; the left side appeared to be quite motionless. From this experiment, it would appear, that if oxygen is not inspired, and if the chemical change in the blood is suspended, obstruction to the circulation through the lung takes place and asphyxia results, although at the same time the mechanical action of the organ may continue. In what way then are we to suppose that the inspired air acts on the circulation ? If we suppose it to act as a stimulus on the capillaries of the lung, the theory is open to objection, inasmuch as it has not been proved that these vessels are capable of contraction. On the other hand, we cannot suppose that the air acts on the vessels of the lung as a sedative. The only conclusion we can come to, in the present state of our knowledge, is, that atmospheric air, applied to the blood in the lungs, promotes its passage through these organs in a manner wholly inexplicable. The motion of the blood through the lungs appears to be determined by a cause independent of any impulse from the containing solids. This view of the question appears to be borne out by what is observed in vegetables and the lower tribes of animals, in which the fluids move without any contraction on the part of their containing solids, and seems to be confirmed by the vibratory motions of the ciliæ, as observed by Purkinje, motions which Dr. A. seems inclined to attribute to *spontaneous currents in the fluid itself.*"

Harvey (we believe) and others maintained, that the blood itself had a power of locomotion ; so that even here, there is no novelty in the professor's notions. But we confess that we are not converts to this doctrine, however highly supported. We are more inclined to think that fluids move by some impulse unknown to us, than that they move *spontaneously.* We think it is very far from being proved that the sap ascends in vegetables, without any *propelling* or *attracting* power, independently of its own spontaneous disposition. It is not unreasonable to suppose that the varying states of earth and air, in respect to heat, moisture, weight, &c. may have a considerable influence on those vessels through which the sap moves. In respect to the vital fluids of the human body, we cannot bring ourselves to believe them possessed of voluntary power of locomotion. If the absorbents have the power of *selecting* the matters which they take up, may they not have the power of attracting and moving them ? If water ascends in a capillary tube of glass, that affords no proof of spontaneous power of ascent in the water. Why does not water ascend when the tube is *not* capillary ? This proves that the size of the tube has more to do with the ascent of the water, than any inherent power of locomotion in the water itself.

Dr. Granville, Dr. Clendinning, Dr. Harrison, Dr. Graves, and others, took part in the debate ; but nothing particular was elicited.

### On partial Amputation of the Foot. By Mr. Whatton, of Manchester.

The reading of this paper appears to have excited great attention in the section. We suppose that but a very brief abstract of it is given.

" Mr. Whatton said, that as far back as 1811, during the Peninsular war, his attention had been drawn to this subject. At that time, when the bones and soft parts of the foot were injured by balls or fragments of shells, the usual practice was to amputate transversely, either at the tarso-metatarsal union, or higher up at the astragulo-scaphoid and calcaneo-cuboideal.—Since he had been appointed to the infirmary at Manchester, he adopted a different mode of operating, which was attended with very superior advantages. He had adopted this plan after a careful study of the relative anatomy of the foot, and was not aware that there was any such operation on record. He tried the operation in a great number of cases, and found it to answer extremely well; of this he hoped he should be able to convince the meeting, as he had an opportunity of showing a patient on whom the operation had been performed, and who was able to walk twenty miles a day.

Mr. Whatton then took a review of the history of partial amputations of the foot from the time of Garengeot down to the present day. The operation of removing a part of the foot had been practised by many before him. The operation then fell into disuse, and continued so until 1789, when it was taken up with some modifications by Chopart, and afterwards further modified by Dupuytren and Richerand. In 1814 this operation became generally known in London, by the publication of M. Roux's work. A method of partial amputation of the foot had been proposed in England as early as 1758, by Mr. Sharp, a similar one by B. Bell, at Edinburgh, in 1778, and the subject had been also noticed by Mr. Hey of Leeds. In 1815 Lisfranc revived Garengeot's operation. To all these different modes of operating one common objection holds, the arch of the foot is destroyed, the patient has no support except what he derives from the heel, and the limb is in fact very little better than a wooden leg. Where the flap is taken from the sole, there is a constant risk of sloughing; and the division of the plantar vessels frequently leaves the flap without a sufficient supply of blood to maintain its vitality. The power of the muscles of the calf is constantly drawing the os calcis upwards and backwards; while the antagonism of the muscles is destroyed by the division of the extensor tendons. In the operation which he (Mr. W.) was about to propose, all these inconveniences were avoided; the spring of the foot is preserved, and if the patient be provided with a proper shoe, scarcely any deformity can be observed.

To illustrate this operation, Mr. Whatton read the details of the two following cases.—The subject of the first case, a man about thirty years of age, was admitted at the Manchester Infirmary, on the 25th of November, 1833. Twelve years before, he had got a swelling of the foot, which was followed by necrosis of the internal and external metatarsal bones. Several fistulous ulcers had formed on the dorsum of the foot, and a probe, introduced into one of these, passed under the integuments, to the extent of four inches, upwards and downwards. He had quick pulse, emaciation, night sweats, and hectic.

Finding that all ordinary modes of treatment had proved ineffectual, Mr. Whatton decided on the longitudinal operation, which was performed in the following manner. An incision, commencing at the root of the fourth toe, was carried, in a slightly curved direction, towards the extremity of the fifth metatarsal bone, and terminated near the outer malleolus. This incision was made on the plantar surface of the foot. A similar incision, commencing and terminating at the same points, was carried along the dorsum. The flaps being dissected off, the knife was carried between the two outer metatarsal bones, down to the cuboid. The outer edge of the os calcis, being found diseased, was also pared off with the scalpel. The second incision removed the next toe and its metatarsal bone in a similar manner, leaving three toes with their corresponding tarsal bones. There was considerable hæmorrhage after the operation, and it was thought advisable to defer dressing the foot, until the patient was placed in bed. The wounds healed kindly, and the man was discharged about twelve weeks after the operation, perfectly well. A cast of the foot was taken ten months after the operation; this shews some fulness about the integuments of the tarsus and metatarsus, but in a cast taken twenty months after the operation, a manifest improvement is visible.

Mr. Whatton here exhibited the casts, which he stated he should feel great pleasure in presenting to the Royal College of Surgeons of Dublin. The patient operated on was exhibited to the meeting.—He walked up and down, with as much ease as a person who had the perfect use of his limbs; and on being required to stand on the leg, singly, he made the attempt in such a manner as to shew that he possessed a considerable power of balancing himself. Much approbation was manifested by the meeting on the occasion.

The next case was that of a female, who had two fungous ulcers of the dorsum of the foot, attended with caries of the heads and shafts of two of the cuneiform bones. There was a constant discharge of fetid, ill-conditioned pus; the patient got no sleep, and suffered very much from pain of the foot. The first incision commenced at the scaphoid bone, and was continued down to the root of the second toe. The second incision, commencing at the same point, ran along the edge of the second metatarsal bone, including the whole of the diseased part. The knife was next passed between the second and third toes, and the metatarsal bones being pressed down, the ligaments were divided. The scaphoid bone, being found diseased, was also removed. The hæmorrhage was easily restrained, and only two ligatures were necessary. The wound healed tolerably well, and the woman was made an out-patient, about two months after the operation.

Mr. Whatton stated, that it was his intention to follow up the subject, and bring it again before the section. He observed that an accurate knowledge of the anatomy of the foot was essential for the proper performance of the operation. He submitted his communication with diffidence, and threw himself upon the kindness of the meeting."

Much approbation followed the reading of this paper, in which Dr. Granville, Mr. Carmichael, Dr. Jacob, Mr. Jefferay, and others, heartily joined. The thanks of the meeting were unanimously voted to Mr. Whatton.

### Dr. Stokes on the Diagnosis of some Diseases of the Thorax, in which there is an Accumulation of the Product of Disease within that Cavity.

Most of our readers are aware that he had previously published two communications in the London Medical and Surgical Journal, on the subject of this paper.

"Without entering on the question, as to whether organic diseases of the thoracic viscera are followed in all cases by some alteration in the volume of these organs, we may divide cases of thoracic disease into two classes; first, those in which there is no manifest alteration; and secondly, those in which there is a manifest alteration of volume. This division, however, is merely arbitrary. These enlargements are of two kinds; either an actual increase of volume of the parenchyma of the lungs, or a distention of its serous covering. The affections in which these occur, may be termed diseases of accumulation. Another, and more important division, is that founded on the effect of disease, in increasing or diminishing the quantity of air within the thorax. If we take empyema on the one hand, and dilatation of the air-cells and pneumothorax on the other, we find that these diseases of accumulation may occur with a diminution or an increase in the quantity of contained air, so that the diagnosis depends, first, on the evidence of accumulation, and next on the physical properties of the accumulated matter. In empyema, there is accumulation and pressure from a non-elastic fluid; in emphysema and pneumothorax, from an elastic medium. In empyema we have, in addition to signs of displacement, proofs of a diminution in the quantity of contained air; in the other affections, we have also displacement, but the quantity of air is increased.

There are, however, some very interesting points of difference, connected with the results of these diseases on the walls of the thorax. In empyema, the dilatation is most remarkable in the inferior portion of the lung; in emphysema, in the superior. A still more remarkable difference appears to be connected with the effect of these two diseases on the muscular parietes of the chest. In empyema, the muscular parietes of the chest yield, in a very obvious manner, to the effects of the disease; the intercostal spaces are obliterated, the affected side enlarged, and the diaphragm depressed. But in emphysema, the disease may be carried to a great amount, without producing these appearances. Now what is the cause of these remarkable differences? To explain this, was the object of the present communication.

It would appear that the explanation of the dilated state of the intercostal muscles was to be sought for in the circumstances attendant on pleuritic inflammation. It is a well-established fact, that when muscular structures are in close connexion with inflamed tissues, their functions become impaired; and in such cases we observe, first an increase, and afterwards a diminution of innervation. In the first place, we have pain, spasm, and irritation; in the second, weakness and paralysis more or less complete. Under the latter condition, the muscular fibres lose their contractility. Dr. Stokes here referred to the researches of Dr. Abercrombie on Ileus, in which the morbid appearances were found to be confined to the dilated and not to the contracted parts. With respect to the evidences in favour of the opinion, that the displacement of the thoracic muscles was the result of paralysis, he stated, that in the first stage of pleuritis we have pain on inspiration, without protrusion of the intercostal spaces; but in the more advanced periods, pain is absent and respiration more free, but the intercostal spaces yield, and we have smoothness of the side produced. The latter circumstances corresponds with the minus degree of innervation, or paralysis. The next evidence is, that mere pressure is not sufficient to produce this. If we examine emphysema or hydrothorax, we shall find that in both there is strong pressure exercised on the muscular parietes of the thorax, as shewn by the enlargement of the chest; yet the intercostal spaces are not necessarily dilated. The last point of evidence is the sudden yielding of the diaphragm, which Dr. Stokes had observed in certain cases of empyema. This yielding was as extensive as it was sudden, and was not accompanied by evidences of increase of effusion. He thought, therefore, that he was borne out in the conclusion, that the protrusion of the intercostal spaces and the depression of the diaphragm are the result of a semi-paralysed state of these organs.

This principle (if established by future investigations) might be applied to the investigation of other forms of thoracic disease. Of pleuritis he had already spoken; it would be necessary to make a few observations with respect to bronchitis and pericarditis. In all these the suffering of the muscular tissue in the first stage has been recognized, but the effects of inflammation in the advanced condition had been neglected. In bronchitis it is a question how far the paralysis of the circular fibres of the bronchial tubes, may account for the accumulation in those tubes which is so commonly followed by asphyxia, in cases of bad catarrhal fever. In such cases we often see the patients dying from the effects of the accumulation in the lung, although there is no remarkable general prostration, and the individuals possess a considerable degree of muscular strength, so far as the system of animal life is concerned. Again, it might be inquired, how far, in dilatation of the bronchial tubes, this condition may have existed. All writers seem to have acknowledged that Laennec's explanation of this occurrence is imperfect and unsatisfactory. Lastly, in cases of pericarditis, this principle (if admitted) would serve to explain the fatal termination of the disease. How accurately do the symptoms of the advanced stage of pericarditis, the syncope, the weakness, the failure of the pulse, correspond with a more or less paralysed condition of the heart? How singularly do the phenomena of the first stage correspond with the increase of innervation which we know to be the first effect of inflammation of the muscular structure? The same principle would serve to explain the supervention of active aneurism on the advanced stages of pericarditis. In the weakened state of the heart it yields to the pressure of the blood, and by degrees its cavities become distended. On the hydrostatic principle the force of this distention must be every moment increasing, and, of course, the progress of the disease will be proportionally rapid. Now, suppose that the inflammatory process ceases; the muscular fibres of the heart recover their tone, but they have an increased duty to perform; and from the well-known law in physiology their growth is increased. To dilatation is added hypertrophy, and thus active aneurism is established. This theory differed from that of Andral, who assumes that the hypertrophy takes place from the first, and omits the possibility of an intervening paralytic condition of the heart.

The last point to which Dr. Stokes referred, related to the phenomena of respiration in Laennec's emphysema. If we take two cases of disease of accumulation, as for instance empyema and dilatation of the air-cells, and suppose that in one the chest yields *pari passu* with the enlargement of the lung, while in the other it is rigid and unyielding; it is plain that the physical conditions and signs must be different. This appears to afford an explanation of the feebleness of respiration in dilatation of the air cells. If the quantity of air in the lung be so great as to keep it forcibly distended even after expiration, it is obvious that on the next inspiratory effort, the volume of air which enters will be minus the expansion of the lung from its distending force. If this be true, and this cause be the principal source of the feebleness of respiration, it should follow, that if the chest yielded easily to the enlargement of the lung, the disease would occur without the characteristic sign; in other words, the feebleness of respiration would be more a measure of the compression of the lung than a direct sign of Laennec's emphysema. Dr. Stokes brought forward an illustrative case, and concluded by stating, that these observations and suggestions should be tested by future investigation."

We are much surprised to find that this important communication led to no discussion. Our experience in medical meetings, however, might have prepared us for this; for we have often seen the best communications pass *sub silentio*, while papers, not worth a farthing intrinsically, have brought forth discussions of the most valuable nature. We think we could account for this seeming anomaly; but we have neither time nor space for speculations at present.

### On the Treatment of Purulent Ophthalmia in New-born Infants. By Dr. Evory Kennedy.

"Dr. Kennedy said that he wished it to be understood by the meeting, that the object of his communication was merely to illustrate some disputed points in practice; on such a subject nothing new or original was to be expected. Purulent ophthalmia was of very frequent occurrence; many cases of it were to be met with in lying-in-hospitals; it was a disease of a violent character, and perhaps caused more blindness than any other affection of the eye. He did not intend to enter into the history of the disease, nor would he stop to examine the question as to its phlegmonous or erysipelatous nature. A great deal of difficulty attended the investigation of the origin of purulent ophthalmia, as connected with a specific virus. As far as his experience went, the proportion of cases which could be distinctly referred to gonorrhœa, or to the leucorrheal discharge, was very small. He had, however, observed that ophthalmia, generated in this way, was of a bad and obstinate character; five of the worst cases he had seen had been produced by infection, and in one of these there was extensive sloughing of the cornea. The disease was observed in the lying-in hospital to commence either immediately after birth, or in a few days afterwards. It was also seen to follow exposure to cold and irritants, a circumstance which goes to prove, that irritation, whether specific or not, may produce it. Viewed without reference to any theory, the disease seemed to consist in a violent and rapid inflammation, speedily followed by a copious secretion from the diseased part. A very remarkable change took place in the secretion of the conjunctiva; this, however, was not peculiar to that membrane; an analogous change was frequently observed in certain affections of the mucous membrane of the genito-urinary and respiratory systems.

With respect to the treatment of purulent ophthalmia in children much difference prevailed. Some treated it with sedatives, others with stimulants; a third class restricted the use of sedatives to the early stage of the disease, and then had recourse to stimulants and astringents. He would proceed to state those means which he had found most efficient. One of the first and most necessary steps in the treatment was the application of leeches. One of these was applied to the inflamed lid, or to the temple in the immediate vicinity of the eye; the former situation was, however, generally preferred. Dr. Kennedy had never seen any inconvenient or alarming hæmorrhage from the use of leeches under such circumstances, and conceived that the extravasation of blood in the loose cellular tissue of the lids might, by its pressure, have some effect in preventing the hæmorrhage. In bad cases, where the inflammatory symptoms ran high with copious purulent discharge, and a tendency to eversion of the lids, the leech was applied a second or even a third time, or oftener. Leeching was not found necessary in all cases; in the milder ones, fomentations, alterative aperients, and the use of a solution of nitrate of silver, removed the disease in two or three days. With respect to leeching, he had to observe that he had never seen any of the bad effects attributed to it by some practitioners; in some of the cases which he had under treatment, a leech had been applied four, five, or even six times to the same individual with benefit.

After leeching, the common practice is to have recourse to fomentations, aperients, and astringent collyria. Dr. Kennedy did not think this a mode sufficient; to treat the disease with effect, it was necessary to produce an altered action in the diseased parts.—For this purpose nitrate of silver seems to be better adapted than any other substance; he had tried it extensively, and could bear ample testimony to its value. He had always employed a strong solution, having found that under five grains to the ounce, it produces little or no effect. Solutions, varying in strength from ten to twenty grains, or even half a drachm to the ounce, were applied to the eye, three or four times a day, and succeeded in effecting a cure, where weaker ones had failed. In some cases, the solid nitrate of silver was applied all over the inside of the lids. This was followed by considerable pain, and a puffing of the lids, which continued some hours after the operation, but was easily removed by sponging the eyes with cold water. In obstinate cases, besides leeching and the nitrate of silver, alterative aperients were employed. Scarification of the lids was not resorted to in any case, and Dr. Kennedy thinks, that in the early stage it is objectionable. A close and constant attention to cleanliness was found to be of the greatest use, and he had observed that those nurses, who were careless in washing the eyes of the children after birth, had the greatest number of cases in their wards. The foregoing treatment proved ineffectual, where attention to cleanliness had been neglected. His attention was drawn to this circumstance by observing, that all the cases which were under one particular nurse recovered rapidly, and cases which had been going on badly with others, began to improve when placed under her care. On inquiring into the cause of this, he found that this woman kept the child almost constantly in her lap, and removed the discharge with a soft sponge, as fast as it formed. This was also noticed in the convalescent cases. Where the children had been removed from the hospital, a slight discharge still continuing, they generally relapsed from neglect. The case of sloughing of the cornea was one of this description.

The success attending this practice was seen in the rapid subsidence of the disease. On the second or third day, the infant was able to open its eyes, and the worst cases yielded in ten days. Where the disease was protracted, owing to local or constitutional debility, the muriated tincture of iron was given in the breast milk, and occasionally the vinum opii was dropped into the eye."

Dr. Beatty having the charge of a similar institution, could confirm most of the above statements. In the great majority of instances he was unable to trace any specific virus. His experience differed from that of Dr. K. in respect to leeches. The cases did as well without local bleeding as with.

"His treatment consisted at first in the use of cold applications to the eye, constantly repeated, and the use of alterative aperients, and afterwards of the saturated solution of the subacetate of lead, as recommended by Dr. Jacob. If no improvement followed the use of the liquor plumbi, in two or three days, he then had recourse to the solution of nitrate of silver, of the strength of ten grains to the ounce or more. He had found the five-grain solution quite inefficient."

Mr. Byrne, on the other hand, said he had been able to trace every case to a specific infection. He had frequently applied the solid nitrate of silver to the conjunctiva, with great benefit. Swellings of the lids were easily removed by bread and water poultices.

Dr. Collins used leeches with benefit. The nitrate was also beneficial.

Dr. Ireland had seen the disease where there could not be the slightest suspicion of gonorrhœa. He never observed any bad consequences from leeches. He applied the leech to the conjunctiva lining the under lid. After the inflammation was reduced, he applied a solution of the nitrate—20 grains to the ounce of distilled water—with benefit. The disease lasted longer in his hands, than in the hands of Dr. Kennedy or Dr. Beatty.

Case of successful Cæsarean Operation. By G. B. Knowles, Lecturer at the Manchester School of Medicine.

"The patient after being delivered of her fourth child, received an injury, which was followed by pain of the hip-joint, and loss of power in the lower extremities. Since the commencement of this disease she had several miscarriages. In November last she was again seized with labour-pains, and on examination, it was found that the sacrum projected in such a manner as to feel like the head of the child, narrowing the lower outlet of the pelvis to two inches by one. The operation was performed thirty hours after the commencement of labour, and six after the rupture of the membranes. Though the incision was made over the placenta, very little blood was lost, and the patient bore the operation extremely well. With the exception of tympanitis, she had no bad symptom for the first two days; on the third she had vomiting and hiccup, which yielded to treatment, and the tympanitis was removed by the use of turpentine. Owing to the distention of the belly, the lips of the wound could not be brought into apposition until the fifth day; in the interval, a limpid fluid was discharged from the opening. The patient recovered in about a month."

### Dr. Corrigan on the Bruit de Soufflet.

"The sound to which bruit de soufflet has been given, is produced in various parts of the circulating apparatus. Its existence has been ascertained within a comparatively short period, and is due to the inquiring spirit of modern investigation. Few things are more interesting, as objects of pathological curiosity, than the production of sounds in the vessels of the human body under certain circumstances. The nature of these sounds has been examined with all the attention which the subject deserves, and not only has their existence been determined, but it has been found that they constitute some of the most important signs of disease.

It is interesting to inquire, on what peculiar mechanism bruit de soufflêt depends, as unless we are properly acquainted with the manner in which it is produced, we can never apportion to it its due importance, or estimate its proper value as an indication of morbid change. The first part of this communication I shall not read; it consists of an analysis of the various opinions of others, as to the mode in which this sound is formed. I shall merely state, that Laennec supposed it to arise from spasm; and to Dr. Williams, who has followed him in the same path of inquiry, we owe the suggestion, that it might be found to arise from the operation of physical causes. Dismissing the examination of these and various other opinions, I shall proceed at once to the statement of my own views on the production of this sound, remarking *in limine*, that it is heard under a great variety of circumstances. We hear it in narrowing and in dilatations of the aorta, in narrowing of the ventricular opening from disease of the valves, and in permanent patency of the aorta, in varicose aneurisms, in aneurismal varix, in the vessels of the uterus during pregnancy, and even in vessels without any appreciable disease. For the production of bruit de soufflêt the simultaneous presence of the two following conditions are necessary:—first, an irregular current-like motion of the blood (instead of its natural equable movement), tending to produce corresponding vibrations on the sides of the arteries or cavities through which it passes; and secondly, a state of the arteries or cavities themselves, by which, instead of being kept in a state of tense approximation on their contained inelastic blood (and which would necessarily prevent any vibration in their sides), they become free to vibrate from the play of the currents within on their parietes, and by these vibrations give to the sense of touch 'fremissement,' and to the sense of hearing 'bruit de soufflêt.'

If you press on the femoral artery below Poupart's ligament, so as to diminish the calibre of the vessel, you necessarily diminish the supply of blood to the artery below the point of pressure, while the outlet through its branches continues as before. You do not interfere with the action of the heart above or the artery below, you merely diminish the area of the vessel at the part where pressure is applied. Now if a finger be placed on the artery, a short distance below the point of pressure, a fremissement is felt, and if the stethoscope be applied over the same spot bruit de soufflêt is heard. This sound is present in a very remarkable degree in narrowing of the auriculo-ventricular openings of the heart. In this disease the free edge of the valves is most commonly the seat of morbid action, it becomes thickened and drawn in, and thus narrows the opening. Now the ventricle after each contraction leaves its sides in a flaccid state, favourable for being acted on by the next gush of blood from the auricle into the ventricle. The consequence of this is, that the fluid, passing through the narrowed auriculo-ventricular opening, is, in obedience to a well-known law in hydraulics, thrown into diverging currents, and if the hand be applied to the chest, a fremissement may be felt, and a loud bruit de soufflêt heard.

Having mentioned the occurrence of bruit de soufflêt in the narrowed state of an artery, as also in narrowing of the auriculo-ventricular openings, I shall contrast with those a peculiar condition of the aorta, viz. permanent patency of its mouth, in which the sound is heard without any narrowing whatever. [Dr. Corrigan here exhibited drawings of the disease in question.] In some of these cases, the semilunar valves have perforations or holes in them; in others they are thickened and bound back to the sides of the aorta; in others they are ruptured. In some instances, however, the valves remain healthy, the mouth of the artery becoming dilated, so that they cannot close across its mouth; and in these instances, how is this sound produced? It arises from the artery not admitting, in these conditions, of being kept in a sufficiently tense state, so that at the next rush of blood the blood sent in does not move equally, and this current-like motion of the blood playing on its sides produces in them corresponding vibrations, and the sound is heard.

I have noticed all these cases to shew under how many various and seemingly contradictory circumstances it may occur. During pregnancy it may be distinctly heard in the vessels of the uterus after the fourth or fifth month. If we examine the state of these vessels, we shall find that the conditions necessary for the production of bruit de soufflêt are present. Their free anastomosis with veins and sinuses permits them to become partially flaccid in the intervals of the heart's contraction, their sides are thin, and the rush of blood into these comparatively flaccid tubes at the next contraction of the ventricle, gives rise to the current-like motion on which the sound depends. The existence of similar conditions will explain its occurrence in varicose aneurisms and aneurismal varix.

Having alluded to those cases in which it is heard in certain diseased conditions of the heart and arteries, I may notice those cases in which its occurrence is unconnected with vascular disease. If a patient be blooded too much, or if an animal be dying from the effects of hæmorrhage, this sound is heard in the heart and great vessels. Here, in consequence of the quantity of blood which has been abstracted, the equilibrium of the circulation is destroyed, and the arteries not having a sufficient quantity to keep them in a tense state, bruit de soufflêt is the consequence. We also meet with it occasionally in the healthy state of the heart, in nervous and irritable individuals. In this case the equilibrium of the circulation is destroyed by various causes of excitement, and the calibre of the vessels becomes disproportioned to the quantity of contained blood, so as to give rise to a certain degree of flaccidity of their walls. It is a well-known observation, that this sound is never heard in plethora or inflammatory fever, for in these conditions of the system, there is not room for the vibrations of the arterial tunics.

Dr. Corrigan concluded, by detailing an experiment in proof of the foregoing theory.

A small bladder in one instance, and a length of gum-elastic tube or gut in another, were interposed between two cocks, the upper connected with a water cistern; the cock at the other or lower end being the discharging orifice of the bladder or gut. On allowing the water to flow through, the sound of bruit de soufflêt and the sensation of fremissement were perceptible in the intervening bladder or tube, until (from the upper pipe pouring in fluid faster than the lower discharged it) the bladder or gut became tense, and then both sensations ceased, the discharge of fluid from the lower pipe continuing all the time. This experiment was applied to explain the occasional presence and absence of bruit de soufflêt in aneurisms; the sound being present in an aneurism, if the parietes can from any circumstance become at all flaccid in the interval of the heart's contraction, and being absent where the parietes are distended and tense."

The thanks of the meeting were voted to Dr. Corrigan by Mr. Crampton. Dr. Harty had observed bruit de soufflêt in all the arteries where a polypus of the left ventricle of the heart existed. Dr. Williams observed that his views had not been stated quite correctly by Dr. Corrigan. He had attributed the bruit de soufflêt to narrowing or obstruction of the vessels; but he did not deny that circumstances might modify the course of the blood, and have a share in producing it. He thought Dr. Corrigan's explanation would not apply to ossified aorta, in which the bruit is sometimes heard. But Dr. C. was able to defend his doctrine against all objections.

" In reply to Dr. Williams' observations he would say, that he was aware of the existence of the bruit in ossification of the aorta. In such cases, when the first current has been thrown back, the next impinges on a portion of the tube most likely to be thrown into sonorous vibrations. This circumstance had been already noticed by Dr. Wm. Stokes, and he had remarked, that where this intense musical sound occurs, you may guess fairly that the disease is ossification of the aorta."

A paper was then read by Dr. Perry, of Glasgow, on typhus; but no particulars are published. An ingenious curved drill catheter was shewn by Mr. L'Estrange, when the meeting was adjourned.

*Fifth Day, August 14th.*

Dr. O'Beirne read an abstract of his views respecting "defæcation;" but, as we dedicated a long article to his work in a late Number of this Journal, we need not dwell on it here. We think it rather injudicious for authors to hash up published works on such occasions as the present. Dr. O'Beirne, like all *doctrinaires*, is more and more convinced, by every day's experience, of the justness of his views. He still maintains that the rectum *is not the receptacle for fæces*, and that the sigmoid flexure of the colon is never found in the left iliac fossa, except when distended by fæces. When empty, he maintains that it lies in the pelvis, "doubled over the rectum."

"From the results of his numerous experiments and pathological investigations, Dr. O'Beirne comes to the conclusion, first, that the ileo-cœcal valve permits the fæces to pass readily into the colon, but prevents their return into the small intestines; secondly, that the cœcum and colon, from their position and relations, must be always more or less distended with fæcal matter; thirdly, when these become so distended as not to be capable of receiving any more, the small intestines must become also distended; fourthly, that fæcal accumulations are never found in the rectum, except in cases of injury done to the nervous centres, followed by paralysis, and in very weak individuals of a sedentary habit."

Researches on the Effects of Cold and Climate, with a Mode of measuring the Degree of Refrigeration in cooling Bodies. By Dr. Osborne.

"It would be interesting (said Dr. Osborne) to ascertain, by a tabular view, the proportion of diseases produced by cold. To arrive at some information on this subject, I caused the patients in Sir Patrick Dun's Hospital to be examined some time ago, and found that out of fifty-seven cases of various forms of disease, thirty-four were attributed to cold. In these, the cold was contracted in the following manner: twelve from damp clothing, five from wet feet, three from bathing, fourteen from exposure to cold currents of air while heated.

It is unnecessary for me to dwell on the importance attached to the investigation of the nature and effects of this powerful agent. The temperature of the human body, which may be estimated at 98° of Fahrenheit, is so equally and uniformly diffused, as to secure to all parts a nearly equal degree of warmth; and this has been further regulated by means of clothing, so as to enable man to adapt himself to the diversity of climates and seasons. Thus the Esquimaux of North America, and the inhabitant of central Africa, are equally intent on maintaining an uniform degree of temperature. In defending the cutaneous surface from the injurious effects of cold, man had been eminently successful; but there is a very extensive surface which he cannot protect; he cannot prevent it from coming in contact with the vast expansion of mucous membrane in the lung. The expired air is always heated to near the temperature of blood, no matter how cold it might have been when it entered the lung; and if we reflect that a quantity of air, equal to about twenty-eight cubic feet, is required every hour for the purposes of respiration, we may be able to form some estimate of the vast quantity of heat which is subtracted from the system in this manner. As all warm bodies give out heat in proportion to their temperature, and have a tendency to continue this process until the surrounding objects are raised to the same standard, it follows that the lung must, under ordinary circumstances, be constantly employed in adding to the temperature of the inspired air. This process, however, appears to go on with varying degrees of intensity, the air being more heated in the first than in the second step of the process, that is in inspiration more than in expiration, and that by the time it has got into the aircells it has acquired more than one-half its heat. What the source of this vast quantity of heat was, it was not easy to ascertain. That it was not dependent on respiration, was proved by the experiments of Sir B. Brodie, who had found, that although, by keeping up artificial respiration, the circulation went on, and the change of colour in the blood took place, still the body cooled down rapidly.

From these and other considerations it would appear, that we have good grounds to look upon respiration as a cooling process, by which, in certain states of the atmosphere, a vast quantity of heat is abstracted from the system. This view of the question leads to many interesting considerations connected with disease. In the sinking energy which accompanies many forms of mucous disease, when the coldness of the surface and extremities shews that the power of generating heat is far below its natural standard, the effects of reduced temperature assume a very important character. The air then receiving very little heat in its passage, reaches the ultimate vessels of the lung in a low state of temperature, the consequence of which is torpor of the capillaries, which are thus rendered incapable of acting on the blood; and on examining the body after death, the right cavities of the heart are found to be gorged with blood. An unfavourable result of the same character may arise from the application of cold air to the surface of the lungs in low fevers, accompanied by coldness of the extremities and diminished vital energy. A knowledge of this principle may serve to explain the greater frequency of deaths by night under such circumstances; it also suggests the propriety of attending to the temperature of the apartments where sick persons are lying, in order to prevent the operation of a cause so likely to prove injurious.

In the healthy individual, the effect produced by the application of cold air to the surface of the lungs is slight, and chiefly limited to the rima glottidis and larynx. This occurs most frequently in passing suddenly from a heated apartment into cold damp air. When the air gets into the minute bronchial tubes, its effects are much less likely to prove injurious; it acquires such a quantity of heat during its transit, that its injurious properties are neutralized. It is a common opinion, that sleeping in damp apartments has a powerful tendency to produce cold. This, however, is found not to be the case, where the residents are careful in keeping their clothes dry. I was informed by a military medical officer, that by adopting a precaution of this kind, he had been able to preserve all his men from the effects of cold, at a time when they were quartered in a damp, newly-plastered barrack. Every individual in health raises the temperature of the bed in which he lies to 80°, and the only way in which cold can prove injurious, under such circumstances, is by being applied to the lung. I come now to consider the effects of cold on the stomach. This organ appears to possess but very little sensibility to cold or heat. We take tea at the temperature of 140°, and ice at the temperature of 32°, without the difference being perceived by the stomach. The impression does not seem to extend beyond the mouth and pharynx, and hence we frequently see persons who have taken very hot substances into the mouth, swallow them quickly to get rid of the sensation of heat. Cold seems to act on the stomach rather as a stimulant than a sedative. This is particularly observed with respect to ice. Many persons will feel thirsty, and drink in half an hour after taking an ice. In some instances the application of cold to the stomach proves highly irritant, giving rise to extensive and violent inflammation; this may be observed in that form of gastritis, which results from drinking cold water while fatigued after violent exercise. This, perhaps, may be explained by supposing that the overpowering influence of the cold produces, not reaction, but that torpor and distention of the vascular system, which, perhaps, is the character of inflammation in the first stage.

Where cold is applied to the surface of the body we observe two different results. Where the application is transient, and the circulation active, cold is followed rapidly by temporary vascularity, with increased heat, and redness. When severe, and continued for a length of time, it produces a paleness and shrinking of the part, followed at first by lividity, and afterwards by tumefaction. Taking these facts in connexion with the dilated state and diminished tonicity of the vessels, as proved by Dr. Alison, does it not appear that cold produces its effects by superinducing torpor of the part, which if not removed by transient reaction, will be followed by the more permanent and vigorous reaction of inflammation?

The various contrivances adopted by mankind to protect themselves against the influence of cold, by clothing, habitation, and artificial heat, prove how much its abstraction affects our comforts. Men of science have explored with assiduity the effects of temperature on the thermometer and hygrometer, and have observed its varieties with great care; but it must be confessed, that hitherto meteorology has contributed little or nothing to our knowledge of the phenomena of health and disease. One consideration has been entirely omitted, and this refers to the cooling power of the atmosphere, as estimated not merely with reference to our senses. The human body is generally placed in a medium colder than itself. The degree of cooling power possessed by this medium has never been accurately measured, and is merely judged of by the sensation of the individual. I beg leave to introduce to the notice of the meeting a mode which I have employed for some time in measuring the degree of cooling power enjoyed by atmospheric air under various circumstances. The instrument which I employ for this purpose consists of a spirit thermometer without a frame, carefully graduated from 80 to 90 degrees inclusive. Having heated this to 90 degrees, it is exposed to the cooling influence of the air in different places, the space of time which the spirit takes in cooling down from 90 to 80, being the measure of the refrigerating power of the air. From several experiments which I have made with this instrument, I find that the cooling power is inversely as the time required to bring the spirit level from 90 to 80. The spirit thermometer is used in preference to one of mercury or water, because it is less rapid in its descent, and affords more time for observation. The numbers 80 and 90 are selected as including the ordinary range of temperature on the exterior of the body. The thermometer I use has a bulb of an inch in diameter, and a stem of six inches. The spirit is made to rise by heating the bulb with the hand or warm water. In order to insure accuracy, it will be necessary to have a number of these instruments (heated to 90) placed in air at the temperature of 60, and to select one of them which shall be found, on several trials, to cool down to 80 in the same given space of time.

The following is a table of the results given by this instrument in different experiments. Heated to 90, and placed in the open air, at a temperature of sixty, the instrument cooled down from 90 to 80 in three minutes, when at rest; when exposed to a slight breeze, it cooled down in one minute forty-eight seconds. In a temperature of 62, it cooled down, at rest, in three minutes; when fanned with paper, it cooled down in one minute and thirty seconds. In a temperature of 68½, it cooled down, at rest, in four minutes; held by an individual walking briskly about, it cooled down in two minutes forty seconds. This shews that the rate of cooling in the same temperature, occurring in a person at rest, and in an individual exposed to a breeze, is in the proportion of five to one. Compare this with the fact recorded by Captain Parry; he found that in the Polar regions, the men could bear the air at a temperature of zero while at rest, but could not when walking about. From the superior refrigerating power possessed by a breeze, it was very probable that if the persons who perished in the black-hole at Calcutta, had allowed one of their number to sit under the window, and fan the rest of the party, their lives would have been saved. The ordinary thermometer cannot measure this effect of the cooling powèr of air put in motion. In places along the sea coast, where the cooling power of the air was very great, it afforded no information; and yet in such places a very appreciable difference will be shewn by the instrument which I hold in my hand. These observations will apply with increased force to the western coast of Africa, and the West Indies. If you look to Thompson's meteorological tables, you cannot find any thing in them, capable of explaining the vast difference, in point of danger, between the temperature of the day and night. This instrument, I think, would enable us to give some explanation of the bad effects connected with these changes, which are totally inappreciable by the common thermometer."

### Dr. Hutton on Congenital Defects.

"Dr. Hutton read a long paper detailing a remarkable case of deficient development and malformation. We greatly regret that our limits will not permit us to give even the substance of this paper. Yet we cannot pass over this paper entirely unnoticed. The subject was an ideot, aged 31 years, who died in the Richmond Hospital of diffuse inflammation of the mucous and submucous membranes of the pharynx, larynx, &c. There was a remarkable deficiency in the right hemisphere of the brain, though the head presented no particular deformity externally. The brain, generally, was small, and the skull much thickened, particularly in the frontal region. A great part of the right hemisphere was deficient, and a serous cyst, filled with limpid fluid, five inches in length, and nearly three in its transverse diameter, occupied the hiatus. The brain was so soft from putrefactions, after the drawings had been taken, that no minute examination of it could be made.

Dr. Hutton said, that he (the deceased) seemed to have very few ideas, and those of the simplest kind, principally connected with his sensations. He apprehended some ideas readily, but could not give them utterance, had little use of language, and articulated indistinctly. He had strong local and personal attachments, was very fond of his nurse and attendants, and frequently asked leave to go and see them while in hospital. He was inoffensive in his manners, well conducted, and of a cheerful disposition. He was not indifferent to money, and asked for his penny like others. During his illness, he frequently cried out to his medical attendant, 'make me live, make me live;' and referred to the throat as the seat of his disease."

### On Aneurism by Anastomosis. Mr. Adams.

This was an elaborate paper, and drawn up with great ability and evidence of practical observation of the subject. We regret that we are unable to give an account of it here. When published, we shall present an analysis to our readers.

The final adjournment being moved by Dr. Harrison, Dr. Alison rose, and said—

"Before the Section adjourns, permit me, on the part of those who are about to return to their homes, to express their warm thanks, and a deep sense of gratitude, for the unvarying kindness and hospitality we have experienced since our arrival. Sir, the hospitality of your city is well known, and the feelings of her inhabitants are as generous as they are enthusiastic; but I must say, in the utmost sincerity, that never have I been so kindly received, never so gratified and amused as on the present occasion. But, Sir, there is another duty which we have to perform, another feeling which we are bound to express in terms no less vivid, in language of equal truth. We have to evince the high respect we do and always shall entertain for the talent, industry, and research, with which every branch of the profession is here conducted, and the great ability and spirit with which the science of medicine is here cultivated."

Dr. Clendinning bore testimony to the truth of Dr. Alison's sentiments, and Dr. Graves addressed the Section on its approaching dissolution.

"I need not in such an assembly say, that I feel deeply gratified in having the honour to second that motion. Gentlemen, before we part, and while those feelings of mutual esteem and good will, those aspirations for the interests of medical science, in which we have all partaken, are still fresh and vivid, permit me to offer one word of advice. Do not omit attending every meeting of the Association. If the profession were to send its deputies in greater numbers to these meetings, such an union of talent and information would be likely to impress the nation at large with the importance of the medical profession to mankind. Without entertaining the slightest feeling of jealousy, I will assert, that among all the sections there is none more important, none more useful, than that which embraces the distinguished individuals which I now see assembled before me. I will state candidly, that I think the medical profession has not had its due weight in the British Association. But when I say this, let me not be misunderstood, I merely wish to stimulate the medical section to increased activity, I merely wish to excite the profession at large, to co-operate with more zeal in promoting the objects for which the British Association was formed, by attending each meeting in greater numbers, and by bringing to the common store a more abundant contribution of important facts, and well founded conclusions. To promote the attainment of so desirable an end, let Dublin, Edinburgh, and London, let the chief towns of all Britain vie with each other in the number and ability of the deputies they send to the medical section, at the next meeting of the Association."

We regret to find so few of our metropolitan brethren at the Dublin Meeting. There is some excuse, however, in the distance, and in the protracted session of Parliament, which kept London full, to the beginning of September, and even till the middle of that month. This was the cause of our own absence, and that of many others. It shall not operate next year, when we anticipate an overflowing concourse at Bristol, where, we understand, the greatest accommodations have been offered by the corporate and other public bodies. We think the medical Section should have evening meetings, for which there is the example of both houses of Parliament. Seven days do not afford nearly sufficient scope for the reading of papers and the necessary discussions. Two or three hours, every evening, should be dedicated to these purposes.

---

### OBSERVATIONS ON THE VARIETIES OF TRUSSES.

Mr. Acret, an ingenious truss-maker of London, has lately published a treatise on hernia, chiefly addressed to the non-professional public. We may safely be excused from noticing its anatomical and surgical portions, but we think we may introduce the observations of a scientific truss-maker, on the various forms, the different uses, and the relative advantages of trusses. The information may probably be serviceable to many of our country readers.

It is rather remarkable how much more frequent hernia would seem to have become of late, were the number of trusses distributed from year to year to be taken as an indication of the comparative increase or decrease of the disease. Fifty years ago, says Mr. Acret, there were only five truss-makers in London, and I believe not one truss society; now there are forty truss-makers, and several truss societies, many of which societies make annual increases to the number of trusses for gratuitous distribution. The City of London Truss Society, in 1818, gave away 227 trusses, in 1830, 4226. How can we account for this extraordinary increased demand for the remedy, otherwise than by the greater frequency of the malady? and yet labour is assigned as the principal cause. It is true this great demand for trusses may be partly accounted for by the fact, that the instruments being now more perfect in their construction and the complaint better understood, it is not so much neglected as formerly, yet this admission can apply only to a small extent."

Mr. Acret remarks that trusses may be classed under the terms elastic and non-elastic, or trusses with and without springs. The trusses he describes particularly are Gawan's Non-elastic truss—the Old Serpent Truss—Egg's German Truss—Cole's Trusses for Scrotal and for Inguinal Herniæ—Salmon's Opposite-sided Truss—and Adams' Graduated Pressure Truss.

I. *Gawan's Non-elastic Truss.*

This is nothing more than a stuffed belt, with a pad and straps. It is not elastic. All surgeons conspire to condemn this as unsafe, and consequently an improper instrument.

II. *The Serpent Truss.*

This is usually called the "Common Truss." It is on the whole a very good one. In its application the pad is placed just above the os pubis, and secured in that situation by the under strap, or what is usually called the thigh strap. Attention to the form of the pad is of the greatest importance, as on it depends the security and comfort of the wearer. To secure the rupture is of course the first consideration, the comfort of the patient the second. Some ruptures are of that description that we are necessarily obliged to use that form of pad which it is painful to wear; others are so easily secured as to leave us at full liberty to choose the easiest form of pad. It is desirable that the pad should be so constructed as to secure the rupture, without subjecting the spermatic cord to pressure. Mr. Acret would say that in every case of external inguinal hernia, where the natural distance of the rings is preserved, a convex pad will be found to answer best, for these reasons, that such a pad most effectually closes the canal and upper opening, and is more likely to produce a radical cure; that less risk is incurred of bruising the cord; that it is less in the way in stooping or sitting; and that it is much the easiest form of pad to wear.

If the hernia be internal inguinal, in which case the contents pass direct from the abdomen without traversing the canal, elevation in the centre of the pad would render it useless, because in this case we require the pressure close to the bone; here then we must apply a flat pad or as is sometimes necessary, one with an elevated circumference. When the case is external inguinal hernia, but the upper ring has approximated towards the lower one, thereby shortening the length of the canal; so far as this has taken place must the convexity of the pad be lessened or removed. In some cases the most secure pad is one that is convex at the extreme end and lower edge of the pad. The difference, in point of comfort, between a flat and convex pad is proved by the frequent complaints made by patients of the former, and the relief they experience when the latter is substituted. Mr. Acret blames the London Truss Society for employing the flat pads far too indiscriminately.

III. *Egg's Patent Truss.*

"Mr. Egg's truss is somewhat similar in shape to the Common Truss; but instead of the spring being made of sheet steel, it is made of bar steel, forged to the shape of oval wire, and drawn out tapering at the end most distant from the pad. The superiority of the elastic spring truss over the non-elastic is, that its elasticity enables it to keep a close though yielding compression on the ring during the alterations of size and different positions of the body; whereas in Egg's truss the spring is so compact that it has very little elasticity, and from the want of this elasticity it is named 'self resisting,' that is, the compression is constant without any relief. To adjust one of these trusses properly the spring should be set to the exact shape of the body. In shaping the common Truss it matters not how near the front pad approximates to the back of the truss, but in Egg's truss the spring is set very much open. From the compactness of the spring it requires great force to open it, it should therefore be so shaped that the distance between the pad and back of the truss is nearly equal to the diameter of the body; for instance, if the diameter of the body, at the groin, be nine inches, then the diameter of the truss should be eight inches, and the same rule will apply let the hernia be large or small, only that a stronger or weaker spring must be selected, and so selected, that when the truss is on the force requisite to open it to the diameter of the body shall be greater than the impetus by which the rupture is protruded. The supposed superiority of this truss is that it has no disposition to press beyond the one inch. Although it is professed that no straps are necessary, yet in many instances the truss is useless without them."

Mr. Acret has seen the parts much bruised by these trusses. He could never wear one himself for more than half an hour. We think, however, he rather over-rates the inconveniences arising from their use. We have known several patients wear Egg's trusses with great advantage, and with little discomfort.

IV. *Cole's Trusses.*

Mr. Cole is the inventor of two trusses; one which he calls his Scrotal Bandage, and the other adapted for Inguinal Herniæ, which have not passed the lower ring. The former, Mr. Acret, strongly condemns, as it offers no provision for adaptation to the occasional difference in the size of the body.

"Mr. Cole's truss for inguinal hernia is certainly a much lighter and more useful instrument than his scrotal bandage. The spring of this truss, instead of being made of sheet steel, as most others are, is made from bar steel forged; and as this description of spring has not that elasticity which will be found in those made of sheet steel, the difference is made up by the introduction of a spiral spring. Some of these trusses are made with a three-quarter spring and some with a half-spring; the former intended to apply on the opposite side, the latter on the same side, but they both require a strap to finish the circumference of the body. The truss has two pads, back and front: the back pad is round, similar to Salmon's, and rests on the centre of the back; the front pad is similar in shape to the Common Truss, the spiral springs are placed on each pad, and causes the yielding resistance spoken of by Mr. Cole."

V. *Adam's Graduated Pressure Truss.*

The objections to this are so strong that we need not particularly describe it.

VI. *Salmon's Self-adjusting Truss.*

This is so familiar to the public and profession that description is unnecessary. Mr. Acret thinks that it approaches nearer to perfection than any other instrument in use. The cases where he has found it fail are, in direct inguinal hernia, especially if only on one side. In persons who have no fall in the back, or when the os sacrum happens to be situated very high in relation to the os pubis, and in persons having a prominent os pubis and flat abdomen. In the first case an under strap is indispensable and cannot be used with the opposite-sided truss; in the second case the back pad will inevitably slide down and the truss fall off; and in the third case the front cushion slips up and allows the rupture to escape.

Such are the trusses most employed at present. Mr. Acret offers the following remarks upon their application to inguinal herniæ.

"If the case is external inguinal hernia and the protrusion has not descended into the scrotum, almost any kind of elastic truss, if well made and properly fitted, will secure the rupture, and in such cases we have full liberty to study the patient's comfort. Salmon's truss made to fit well, will be generally found to be the most comfortable, or one of Cole's Inguinal Trusses may be worn with advantage, or the Common Truss with a convex pad; indeed, in mild cases like these, the patient may make his own choice, selecting that which he thinks will inconvenience him the least, but bearing in mind that, when there is great general relaxation, the pad must be rather large. If the complaint has descended into the scrotum, one of Salmon's may still be applied, only the pressure must be greater, and the point of pressure placed nearer the bottom of the pad, or the Common Truss may be applied, with the alteration in the pad suggested a few pages back. Egg's Truss may also be applied in these cases, if the patient can wear it with comfort. If the case is direct inguinal hernia, a Common Truss, with a flat pad, will generally be found the most secure; but as the protrusion in direct inguinal hernia descends immediately into the scrotum, and great difficulty is frequently experienced in securing it; it is impossible to lay down any decided rule for forming the pad; we must be governed by the nature of the difficulties which arise."

---

A TREATISE ON THE MORE OBSCURE AFFECTIONS OF THE BRAIN, ON WHICH THE NATURE AND SUCCESSFUL TREATMENT OF MANY CHRONIC DISEASES DEPEND; BEING THE GULSTONIAN LECTURES, DELIVERED AT THE COLLEGE OF PHYSICIANS, IN MAY, 1835. By A. P. W. PHILIP, M.D. F.R.S. L. and E., Fellow of the Royal Colleges of Physicians of London and Edinburgh, &c.

If ever unmixed happiness has been the lot of man, in this sublunary scene—in this vale of tears, that man is Dr. Wilson Philip! He has attained the summit of his hopes—and, better still, the zenith of his ambition—the FELLOWSHIP of the temple in Pall Mall East! And well he deserves that supreme honour. None ever worked harder or longer (except Dr. Pearson, who died by studying Greek in his 80th year to *pass* the College) than Dr. W. Philip! We almost envy him his happiness—though not his honours. After 20 years, or more, of descent from the meridian of life, and after having acquired a fair distinction in his profession, it was hardly worth his while to *brush* the dust from the feet of the young and *unlicked* cubs of Oxford and Cambridge, in order to sit at the bottom of the bench, and, with grateful tear in the eye of the ancient neophyte, "adore and burn" at the shrine of the PRESIDENT!

Such is the melancholy and too common picture of human nature, when ambition takes the wrong direction, and the laudable love of fame dribbles down into the paltry vanity of personal titles and distinctions!

Shakespeare tells us that some are born to honours—some acquire honours—and others have honours thrust upon them. Unquestionably the *acquirement* of honours is the most honourable; but this depends on the means. Did the neophyte FELLOW join with his FELLOW LICENTIATES, in their petition to the Legislature for reformation in the College? Not he, indeed! His answer to the application was (he can contradict it if we are wrongly informed), that he did not understand *medical politics!* Oh no; but he appears to have been an adept in medical *policy*. He knew a "hawk from a handsaw"—and he had wisdom enough to perceive that, by throwing overboard the REFORMERS, he would gain a niche in the temple of the CORPORATORS! Well; he has taken his low dark seat in the MAUSOLEUM of the TEMPLARS; and, with breathless haste, has issued forth the heraldic proclamation of his collegiate honours and Gulstonian exploits. The hen makes a great noise when she drops an egg—but SHE brings forth original eggs, and *hatches* original chickens. Not so the Gulstonian lecturer. He has no *new* eggs to lay—and, therefore, he *hashes* up the heads and plucks, the gizzards and the giblets of his dead progeny, as a dainty dish to set before the President and Fellows of the College of Physicians! When the last OLLA PODRIDA came forth—the treatise on "SLEEP and DEATH"—we imagined that, as the "brains were out, the man would die;" but we calculated without our host. We did not reckon on a new incarnation of VISHNOU—a tenth AVATER! Harvey and Gulston must have been veritable Hindoos. They were determined that their troubled spirits should rise annually, to be annually merged in the sea of oblivion! Here we have resuscitated the ghosts of all that have run their brief span of existence for 20 years past! Le Gallois—the French Academy—the frogs—the burning wires run down the spines—the vital functions—indigestion—dyspeptic phthisis—minute doses of mercury—sleep and death—all flit, in some shape or other, before our eyes, in every page of this work. This eternal resurrection of dead matters, and fruitless attempts to re-animate them, again and again, by infusing into them those "vital functions" which have long evaporated into air, is most injurious to Dr. Philip's fame. If he can produce nothing new, he should allow old and effete matters to rest quietly in their tombs. Their annual disinterment can only bring forth the chlorides of satire to correct their fetor.

Dr. Philip seems mortified, and well he may, that his physiological experiments and doctrines have made so little impression on the public! After all the years that have been devoted to establish the "laws of the vital functions," and the hecatombs of rabbits, frogs, and other animals, that have been sacrificed at the shrine of science, so ignorant is the world of these same laws—"*that even our most respectable writers seem hardly aware that they have been made a subject of inquiry.*" Now we cannot help sympathising with the Doctor, in consequence of this almost total neglect of his numerous experiments and elaborate inquiries; but the public will only swallow what it likes of men's lucubrations, and it is needless to repine or remonstrate. Dr. Philip does not take it much to heart, that "the medical scribblers of the day" are ignorant or negligent of his "LAWS;" but "when such men as Dr. Alison and Dr. Henry maintain opinions in direct opposition" to what Dr. Philip knows to be the true orthodox faith in Physiology, then, Dr. P. tells us—"it is time seriously to REVIEW the subject." This is alarming intelligence! Dr. Philip turned REVIEWER, after all his ire against that class of *scribblers*. Thank God, we reviewers are not, like the Fellows of the College, obliged to listen to Dr. Philip's reviews—especially reviews of *his own* books! We shall very coolly pass over the whole of this *hash*, so often set before the reader on former occasions, and now absolutely nauseous—together with the *Philippics* against the two Doctors abovementioned, and who are quite able to defend themselves.

It is not till 80 pages of letter-press are dispatched, that the reader will find any attempt to come at all to the subject which the title-page would lead him to expect. In the third chapter, then, we are told that there is established a sympathy between the brain and the stomach. "Even a piece of bad news will instantaneously destroy appetite"—a piece of information which Shakespeare handed down to us 200 years ago, or thereabouts! As the cause of indigestion, then, may be in the brain or spinal marrow, though the effects are only cognizable in the stomach, it is a great object to discover the diagnosis in such cases. Dr. Philip intimates that he has discovered this said diagnosis, in cases where "some of the most eminent of our profession" had failed, and, indeed, gave a contrary opinion as to the real seat of the disease. This is not far from the PUFF indirect. But let us come to the diagnosis itself.

"The nature of the cases in which the original cause of the disease is confined to the brain, precludes the possibility of deriving the diagnosis from any particular train of symptoms: it must be collected from a review of the whole circumstances of the case; from the nature of the remote causes, both predisposing and occasional, the general course of the symptoms, and the effects of the means employed. I shall enumerate the circumstances which chiefly demand attention, and endeavour more particularly to point out the principles on which the diagnosis must be founded.

When the patient is not of a variable and hysterical habit—when the occasional causes have been of a serious and permanent nature, and the nervous symptoms have not shewn themselves for some time after the first application of such causes—when there is not such derangement in the digestive or other organs chiefly affected as accounts for the severity of the nervous symptoms—when the affections, both of mind and body, are less variable than is usual in what are called nervous complaints, and particularly apt to be referred to the same parts of the body—when there is constantly a more or less general tendency to derangement in the secreting system—when the heart is more irritable and the lungs less free, the nervous symptoms not yielding so readily as usual, the depression of spirits more uniform, and the pulse tighter than we should expect to find it from the other symptoms—when either the recurrence of feverishness, or a sense of chilliness and debility, is more frequent than is usual in nervous complaints—when the constitution seems more affected than usual by the continuance of the disease, the strength on the whole decaying—and particularly when the countenance assumes a sallow colour, and an habitually irritable and anxious expression; when the usual means are not attended with their usual effects, our stomachic medicines being in a great degree powerless, and alteratives producing but a transitory, if any, improvement in the abdominal secretions; when these, or several of these circumstances, are well marked in what are called nervous complaints, I have been assured, by repeated observation, *that they are not to be safely disregarded.*"

We quite agree with Dr. Philip, that these symptoms should not be disregarded, whether the cause be in the head or the stomach; but if the reader has been able to pick up any thing definite out of the foregoing diagnosis, we congratulate him on his penetration. We have not been able to do so—but then we are only the ignoble *scribblers* of the day, entirely beneath the notice of Dr. Philip. It is to be remembered, however, that the author, in one of his almost innumerable writings (for he is the most voluminous *scribbler* now existing in the profession), has laid it down as a LAW, that when organic disease is produced by *indigestion*, the morbid appearances are not to be sought in the digestive organs themselves, but in the brain, heart, lungs, or other distant parts. And now the obscurity is rendered doubly obscure by the present doctrine, that many diseases, apparently of the digestive organs, have their fons et origo in the brain! Dr. Philip is a veritable PROTEUS. Catch him as an eel (no easy job), and he turns into a bullock. Seize the bull by the horns, and he instantly disappears under ground as a rabbit—or hops into the marsh as a frog. It is morally and physically impossible to lay hold of him!

But we shall now come to close quarters with our champion, and see what he is made of. "In the mean time (says he), the nature of the disease will be farther illustrated, by turning the attention to the appearances on dissection." Before a dissection of these cases is given, Dr. P. takes care to tell us that they "had been treated as common nervous and bilious complaints; in which *I* had stated to the other medical attendants, that, notwithstanding there were *no symptoms* referred to the *head*, we should find the *brain* organically affected." This was a tolerably bold prognosis; and the enunciation is as modest as any that could proceed from Ramadge, the consumption-curer—Eady, the friend of the constitution—St. John Long, the quicksilver-extractor — Morrison, the hygeist, or any other medical Hercules of the day! The reader is, of course, prepared for a clear, candid, and *authentic* statement of the cases, both as to symptoms and dissection. We give the statement in Dr. Philip's own words.

"The first case I shall mention is that of Mr. A., who was taken ill while pursuing his studies at Oxford. His case was regarded by the physicians of that city as one of *common indigestion.** His health not improving, he was brought to London, and placed under the care of two physicians well known to the profession here. After he had been in London a few weeks, I was called in, in consultation, and, guided by the circumstances *which have been laid before the College*, expressed my fears of a fatal termination, and stated my opinion, in consultation, that although the *stomach and duodenum* were the organs most prominently affected, I believed we should find the origin of the disease in the *brain;* and on dissection after death, which happened in

* The words marked in Italics have been so marked by ourselves.—REV.

a fortnight or three weeks after I saw the patient, and appeared to be the consequence of inanition, the following appearances presented themselves.

The body was examined by Mr. Walker, of St. George's Hospital. In this and the following dissection, the examination was made about twenty-four hours after death, and the body was free from fetor. The following is his report.

'On opening the cavity of the cranium, the membranes and the brain were found *tolerably healthy*, perhaps rather softer than usual, particularly as regards the cerebellum and base of the brain, which, together with the medulla oblongata and cerebral nerves, appeared reduced to a *pulpy state*; so much so that they would not bear the *slightest handling*.

The viscera in the cavity of the chest presented no unusual appearances; the stomach larger than usual, from distension, and presented that appearance which is called the 'hour-glass contraction' of that viscus in a more marked manner than is usually met with; the pylorus much more vascular than usual, and the duodenum much more dilated, vascular, and attenuated than is natural. The whole of the small intestines were more distended with flatus, and much more gorged with blood, than in the healthy state, and of a very dark colour. The liver, spleen, kidneys, and pancreas, were healthy.'"

Will it be believed that, in a case which is to illustrate a great pathological principle, and which is to impugn the judgment of several physicians, and exalt Dr. Philip's diagnostic powers above them all, *not one single symptom of the* ANONYMOUS *case is stated*, whereby we might be able to judge of the reasons why Dr. P. delivered this inspired prophecy! We have no hesitation in averring, that a more disingenuous procedure (not to give it its proper name) was never ventured on, in puffing pamphlet or advertisement, than is here published to the world—and that as a Gulstonian lecture, delivered before the President and Fellows of the College of Physicians! We defy Dr. Philip—we defy the College—we defy the whole profession, to adduce a more shameless piece of SELF-EXALTATION, or a more gross insult to the other attendant physicians, than is here portrayed.

But we have not done with this case. The membranes and the brain were "tolerably healthy," although the cerebellum, the medulla oblongata, and the *cerebral nerves* were in so pulpy a state that they would not bear the "slightest handling." Now in such a state of brain, and within three weeks of the grave, we ask the profession if it was likely that there were *not* various lesions of sensation, volition, muscular power, and other attributes of sensorial integrity? We are not even told what the affections of the stomach and duodenum were in this case. Are we, therefore, to impugn the professional skill of several physicians, without a tittle of evidence, but the *ipse dixit* of the accuser! We call on Dr. Philip to authenticate this case, by stating the symptoms, and referring to the witnesses of the case. Till that is done, we shall doubt the correctness of the report. We are perfectly certain that the disorganization of brain above alluded to was attended, (if the case had been rigidly and accurately observed) by corresponding symptoms, and that the disorder of stomach and duodenum, whatever it was, did not engross the place of the other phenomena.

The second case in illustration, will excite astonishment in the mind of every man who has been half a dozen years in the practice of his profession. We shall give it in the author's own words, marking some portions in italics, for the purpose of drawing attention to them more forcibly.

"The following case was that of Miss C., which run the same course as the preceding, but was of longer duration, having been protracted for more than two years; and here also the patient appeared to die of inanition. Some surprise was expressed that I should wish the head to be examined, as none of the symptoms had been referred to it. The examination was made by Mr. Earle, and the appearances in the brain corresponded, in a remarkable degree, with those just detailed. The symptoms in these cases, as well as the termination of the disease, had been similar, and we find the chief organic affection of the brain of the same kind, and seated in the same parts. The following is Mr. Earle's account of the appearances :—

'In the head, slight effusion beneath the arachnoid membrane; substance of the brain very soft, particularly the crura cerebri and upper part of the pons varolii, which was quite pulpy. Blood-vessels in the substance of the brain large, and loaded with blood. In the chest, right lung greatly compressed by the narrowness of the inferior margin of the ribs, from old adhesions between the pleura costalis and pulmonalis. *Substance of the lungs firm and hepatized.* Left lung more healthy than the right, but slightly hepatised at its upper part.' This state of the lungs it may be remarked is peculiarly characteristic of a failure of nervous influence, as appears from those experiments in which the influence of the brain was prevented from reaching the lungs. The patient had been subject to *cough and oppressed breathing;* pulmonary symptoms, however, had never been a prominent part of the disease. 'The heart,' Mr. Earle proceeds, '*was remarkably small.* In the pericardium, about two ounces of water. In the abdomen, stomach and duodenum much displaced by the compression of the chest by the stays. Towards the pylorus, the stomach much *thickened and indurated,* the *pylorus hard and contracted.* The duodenum large and flaccid; the mucous surface very vascular, villous, and soft, readily breaking down on the *slightest touch,* and apparently approaching to a state of *ulceration.* Liver *almost of a black colour,* and gorged with venous blood: substance of the liver *hardened.* Spleen and kidneys small, but not unhealthy. Intestines generally of a dark colour, from venous congestion.' "

Here, as usual, we have nominal and unauthenticated cases, as far as the history and symptoms are concerned. We ask the profession, for it is needless to ask Dr. Philip, who is blinded by his hypotheses, whether the organic lesions in the thoracic and abdominal viscera are not infinitely more important than those in the brain—and whether it was not far more likely that the state of the cerebral mass was the *consequence* of the various organic changes in the viscera, than the *cause* of them all? We venture to say that not a single physician or pathologist between the Tay and the Thames will undertake to support Dr. Philip's view of the above case.

We shall not enter upon the principles of treatment, discussed by Dr. Philip. Considering his principles of pathology to be narrow, biassed—and, in short, erroneous, we can have no confidence in any *ratio medendi* founded on such a basis. We shall only observe that mercury and antimony appear to be the panaceæ for this class of diseases, and that if the reader is desirous to have the details of the practice, he need not apply to this publication, for the following substantial reason :—" In my treatise on the means of preserving health, and particularly the prevention of organic diseases, I have entered into the particulars of the treatment, the general principles of which I have here endeavoured to explain."

---

### Subcutaneous Inflammation. By Dr. Elliotson.

I have a case, Gentlemen, to speak to you about which is very interesting; it is that of George Hemmings, who was admitted the 4th of last month, with inflammation of the sub-cutaneous cellular tissue. He is about 40 years of age, of a sallow complexion, and has lived intemperately: he has been occasionally subject to attacks of bilious vomiting during the last two years. It appears from his statement that he caught cold last winter while under the influence of mercury, and that he has not been well since. A fortnight since, he was attacked with rigors, which were followed by heat, pain in the head, and thirst. The left side of his throat became sore, the right side of it was also shortly after affected. He observed in a few days that on the right side of the neck and upper portion of the chest, the skin was red, swollen, and very tender when pressure was made on it. He was bled twice for these symptoms, and leeches and a blister were applied to the left side of the chest, which was slightly indurated, but not redder than natural. These measures did not remove the symptoms, but, on the contrary, they continued to extend, spreading quickly over the shoulders, the inside of the arm, and fore-arm, so that motion was attended with much pain. When pressed on, the redness disappears, but immediately the pressure is removed, it returns. Pressure on the calves of the leg gives him pain, particularly on the left one; several indurated lines can be traced on them. There is pain in the popliteal space, on extending the knee joint, and tenderness of the epigastrium on pressure.

He is troubled with a slight cough, which is attended with expectoration of a frothy mucus. Respiration is sibillant over the whole chest, but more especially on the left side; he experiences pain on taking a deep inspiration. His pulse is 90, and rather full——he has considerable thirst, and his bowels are confined—urine scanty and high-coloured—the tongue covered with a yellow fur, in the middle clear, and moist round the edges—countenance anxious. Such is the report of the symptoms on his admission. I concluded at once that there the sub-cutaneous cellular tissue was inflamed. I ordered the shoulders, arms, and the right side of the neck, to be washed with a solution of the nitrate of silver, and ten grains of the pill hydrarg. to be taken every six hours. The caustic had the effect of stopping the extension of the inflammation. The tonsils, velum, and the adjacent mucous membrane became inflamed, for which he was ordered an opening draught and a gargle.

On the 7th he was much better, the neck was softer and less tender on pressure—the mercury affected the gums slightly.

Up to the 23d there was nothing remarkable occurred; he had occasionally slight rheumatic pains about the shoulders; for these he was blistered and relieved—a tonic mixture was afterwards ordered, and save a slight tenderness and hardness in the calves of the legs, with occasional rheumatic pains in the palm of the left hand, and ball of the thumb, he did well; and on the 19th was reported cured.

This case is interesting from the plan of treatment adopted; you may probably remember, that in one case, similar to the present, which we had a short time since in this hospital, that the nitrate of silver was applied, but it had not the effect of stopping the progress of the disease: the man died. On examination after death, the cellular tissue was found in a pulpy, pappy state; but not more so under the parts where the caustic had been applied, therefore that could not be said to have increased the disease. Besides, the gentleman who applied the remedy had done it improperly and partially, rubbing the skin with the caustic in the stick, not, as it should be, in solution. In the present instance, when properly applied, the effect has been decided and successful. The profession are indebted to Mr. Higginbottom for their acquaintance with the use of this medicine externally. It is unquestionably one of the greatest improvements in the modern history of medicine. Before the use of this medicine was known in erysipelas, cases of attacks of that disease in the head used to distress and annoy me. No matter what means were employed, bleeding, mercury, and cold, seemed equally uncertain in their operations; but, since the introduction of this medicine, I do not dread these cases as I used. I have applied it very many times with the most decided success, and I can confidently recommend its employment. The case just related is a most decided proof of its utility. The man was of weak constitution, and bleeding could not have been resorted to: here was a fearful inflammation going on, and, but for this medicine, would probably have terminated fatally. I have used the nitrate of silver in other cases attended with discharges from the rectum, the urethra, and vagina, as an injection, in one-tenth of a grain in gonorrhœa, and one-fourth in leucorrhœa. It has also, certainly, great power taken internally in various nervous diseases, particularly hysteria and epilepsy.—*Dr. Ryan's Journal.*

